# Transcriptome analysis of *Ganoderma lingzhi* (Agaricomycetes) response to *Trichoderma hengshanicum* infection

**DOI:** 10.3389/fmicb.2023.1131599

**Published:** 2023-02-23

**Authors:** Tiantian Wang, Xiaobin Li, Chunlan Zhang, Jize Xu

**Affiliations:** ^1^Agricultural College, Yanbian University, Yanji, China; ^2^Agricultural College, Jilin Agricultural Science and Technology University, Jilin, China; ^3^College of Landscape Architecture, Changchun University, Changchun, China; ^4^College of Plant Sciences, Jilin University, Changchun, China

**Keywords:** *Ganoderma lingzhi*, green mold, pathway, RNA-seq, *Trichoderma hengshanicum*

## Abstract

Green mold caused by *Trichoderma* spp. has become one of the most serious diseases which threatening the production of *Ganoderma lingzhi*. To understand the possible resistance mechanism of the *G. lingzhi* response to *T. hengshanicum* infection, we examined the *G. lingzhi* transcript accumulation at 0, 12, and 24 h after *T. hengshanicum* inoculation. The gene expression analysis was conducted on the interaction between *G. lingzhi* and *T. hengshanicum* using RNA-seq and digital gene expression (DGE) profiling methods. Transcriptome sequencing indicated that there were 162 differentially expressed genes (DEGs) at three infection time points, containing 15 up-regulated DEGs and 147 down-regulated DEGs. Resistance-related genes thaumatin-like proteins (TLPs) (PR-5s), phenylalanine ammonia-lyase, and Beta-1,3-glucan binding protein were significantly up-regulated. At the three time points of infection, the heat shock proteins (HSPs) genes of *G. lingzhi* were down-regulated. The down-regulation of HSPs genes led to the inhibition of HSP function, which may compromise the HSP-mediated defense signaling transduction pathway, leading to *G. lingzhi* susceptibility. Pathway enrichment analyses showed that the main enriched pathways by *G. lingzhi* after infection were sphingolipid metabolism, ether lipid metabolism, and valine, leucine and isoleucine degradation pathway. Overall, the results described here improve fundamental knowledge of molecular responses to *G. lingzhi* defense and contribute to the design of strategies against *Trichoderma* spp.

## Introduction

*Ganoderma lingzhi* S. H. Wu, Y. Cao, and Y. C. Dai (Ganodermataceae, Agaricomycetes) is a traditional Chinese medicinal mushroom in Asia that has been used for thousands of years (Cao et al., 2012). It has good medicinal, health, and ornamental value (Wagner et al., 2003; Cao et al., 2012). The pharmacological action of *G. lingzhi* is based on its solid immune modulation and immune potential. The main medicinal ingredients include polysaccharides, triterpenes, peptides, proteins, adenosine, which have anti-androgen, anti-cancer, anti-diabetes, anti-hypertension, anti-melanocyte, anti-virus, and other health functions (Sanodiya et al., 2009; Qian et al., 2013; Satria et al., 2019). Currently, the main cultivation methods of *G. lingzhi* are substitute cultivation and basswood cultivation in China, mainly distributed in the Northeast region, the Dabie Mountains, the Southeast coast, and other places (Jin et al., 2016; Zhou et al., 2017). In recent years, the market demand for *G. lingzhi* has increased by 18–30% every year. In 2015, China was the world’s primary producer and exporter of *G. lingzhi*, with 10,000 hm^2^ of *G. lingzhi* yield and 120,000 t of spore powder, accounting for approximately 75 and 30% of the world, respectively (Jin et al., 2016; Ma, 2017; Li et al., 2021). Due to the increase of the cultivation years, some diseases of *G. lingzhi* have also been successively discovered. Among them, *Trichoderma* spp. has the characteristics of wide distribution, more kinds, rapid incidence, and strong concealment (Xu et al., 2019). *Trichoderma* spp. has become one of the most harmful pathogens in the cultivation of *G. lingzhi*, resulting in a decline in the yield and quality decline of *G. lingzhi*, which have caused severe economic losses to growers and restricted the development of the *G. lingzhi* industry (Xie and Tan, 2015; Huang et al., 2018).

Following the emergence of *G. lingzhi*, it is primarily controlled through the use of chemical reagents such as pesticides, which cause severe pollution to the environment and make drug resistance more likely (Fu et al., 2013; Xie et al., 2018). Based on six chemical reagents, Yan (2011) screened the bacteriostasis of different fungicides on edible fungi and *Trichoderma* spp. by preliminary screening, inhibition of spore germination, and mycelial germination tests. A total of 50% hymexazol has a strong bacteriostatic effect on *Trichoderma* spp. and a little destructive effect on various edible fungi mycelia, which can be recommended for production. Luković et al. (2021) conducted identification and fungicide screening tests on 22 isolation of *Trichoderma* strains. In fungicide susceptibility tests, all examined *Trichoderma* strains were found to be highly sensitive to prochloraz (ED 50 < 0.4 mg ^⋅^ mL^–1^) and considerably susceptible to metrafenone (ED 50 < 4 mg ^⋅^ mL^–1^). Hence, metrafenone might also be recommended to control the green mold of mushrooms. In aspects of biological control, among 50 bacterial strains isolated from mushroom compost, *Bacillus subtillis* B-38 inhibited *Trichoderma harzianum* T54 (48.08%) and *Trichoderma aggressivum* f. *europaeum* T77 (52.25%) mycelium growth *in vitro*. In plot trials, the incidence of the plots inoculated with the *Trichoderma* strains and treated with *B. subtillis* B-38 and *B. subtilis* QST 713 presented significantly lower disease incidence compared to the control, and results for disease control and yield harvested were comparable to the plots treated with prochloraz-Mn, indicating that *B. subtilis* B-38 and *B. subtilis* QST 713 could be used as suitable substitutes for chemical fungicides (Milijašević-Marčić et al., 2017; Gea et al., 2021). Although these methods can play a preventive role to a certain extent, the control effect is limited, so screening and breeding resistant varieties of *G. lingzhi* is the most economical and effective way to control green mold. Therefore, exploring the defense mechanism of *G. lingzhi* against *T. hengshanicum* will help to speed up the breeding process of disease-resistant *G. lingzhi*.

With the development of second-generation sequencing technology and molecular biology, many researchers have explored the molecular regulation mechanisms of rice bacterial blight (Sana et al., 2010), *Phytophthora capsici* disease (Fan et al., 2022), *Nerium indicum* witches’ broom disease (Wang et al., 2022), *Phytophthora sojae* disease (Zhu, 2018), apple alternaria blotch disease (Zhu et al., 2017), *Chrysanthemum morifolium* black spot (Liu L. N. et al., 2021), and wheat leaf spot (Ye et al., 2019). Bailey et al. (2013) found that the pathogen (*Lecanicillium fungicola*) and the host (*Agaricus bisporus*) changed the expression of their respective genes during the interaction through transcriptome analysis, which initially revealed the host’s defense response mechanism. Ma et al. (2021) found that the expression level of *LeTLP1* was strongly induced in response to *T. atroviride* infection in the resistant Y3334 by transcriptome analysis and quantitative real-time polymerase chain reaction (qRT-PCR) detection. The function of *LeTLP1* was verified by gene overexpression and gene silencing technology. Compared with the parent strain Y3334, *LeTLP1*-silenced transformants had reduced resistance relative to *T. atroviride*. These findings suggest that overexpression of *LeTLP1* is a major mechanism for the assistance of *Lentinula edodes* to *T. atroviride*. This molecular basis provides a theoretical foundation for breeding resistant *L. edodes* strains. *A. bisporus* brown blotch disease caused by *Pseudomonas tolaasii* infection mainly activates the arginine and proline metabolism, cysteine and methionine metabolism, jasmonic acid (JA) biosynthesis, methane metabolism, phenylpropanoid metabolism, shikimate pathway, sulfur metabolism and signaling pathways, as well as oxidative phosphorylation pathways. Transcriptomics data combined with qPCR verification indicated that 10 differentially expressed genes (DEGs), including *PIP1*, *MET3*, *AGX*, *PAL1*, *GCL*, *LOX 1/3*, *PR-like*, *MYB3R*, *UCR*, and *SDHB*, were the most potential genes involved in the early defense. These results revealed the early defense response of *A. bisporus* against *P. tolaasii* (Yang et al., 2022). However, there has yet to be a report on how *G. lingzhi* responds to the pathogen *Trichoderma* spp. In this study, RNA-seq technology was used to analyze the transcriptome of *G. lingzhi* in response to *T. hengshanicum* infection, which has a great significance for the breeding of resistant varieties of *G. lingzhi* and the prevention and control of soil pollution.

## Materials and methods

### *Trichoderma hengshanicum* and *Ganoderma lingzhi* cultures, and inoculation method

*Trichoderma hengshanicum* “1009” and *G. lingzhi* “11GL-16” were used in all experiments and preserved in the Development and Utilization Laboratory of Fungi Resource of Jilin Agricultural Science and Technology College. *G. lingzhi* was grown in an edible fungus base. For *G. lingzhi*-back inoculation, the fruiting bodies of some growth vigor were strictly selected. *T. hengshanicum* isolated on potato dextrose agar (PDA) was propagated in a constant temperature incubator at 25°C for 4 days. The *G. lingzhi* fruiting bodies were inoculated with 5-mm-diameter mycelial blocks (from PDA culture plates). The control group inoculated PDA agar blocks without mycelium. At 0, 2, 12, and 24 h after inoculation, the *G. lingzhi* fruiting bodies were cut off with a sterile scalpel at a distance of 5 mm from the lesions and stored at −80°C after quick freezing with liquid nitrogen. At 0, 2, 12, and 24 h after inoculation, three fruiting bodies were taken from each replicate of each treatment group. The frozen samples were used for RNA sequencing.

### RNA extraction, library construction, and sequencing

After 0, 2, 12, and 24 h of infection, more than 500 mg of fruiting bodies were collected for RNA extraction. Total RNA was extracted using a Trizol reagent kit according to the manufacturer’s protocol. RNA quality was assessed on an Agilent 2100 Bioanalyzer and checked using RNase-free agarose gel electrophoresis. Illumina MiSeq library construction was performed according to the manufacturer’s instructions (Illumina, San Diego, CA, USA). To separate the mRNA from the total RNA, magnetic beads with poly T oligos were used. Then the enriched mRNA was fragmented into short fragments using a fragmentation buffer and reverse transcribed into cDNA with random primers. The cDNA fragments were purified with QIAquick PCR (Qiagen, Venlo, The Netherlands). Extraction Kit, end-repaired, and A base added and ligated to Illumina sequencing adapters. The ligation products were size selected by agarose gel electrophoresis, PCR amplified and sequenced using Illumina MiSeq by Personal Biotechnology Co., Ltd. (Shanghai, China).

### Data filtering, *de novo* assembly, and gene function annotation

Reads obtained from the sequencing machines included raw reads containing adapters or low-quality bases, which would affect the following assembly and analysis. The clean reads were retrieved after trimming adapter sequences and removal of low quality (containing >50% bases with a Phred quality score < 20) using the FastQC tool. Transcriptome *de novo* assembly was performed with the short reads assembling program-Trinity (Grabherr et al., 2011). Firstly, a short sequence library of K-mer length was constructed using high-quality sequences. Then the short sequence was extended by the overlap of K-mer-1 length between short sequences to obtain the preliminary spliced contig sequences. Next, Chrysalis clusters related contigs that correspond to portions of alternatively spliced transcripts or otherwise unique portions of paralogous genes and then builds Bruijn graphs for each cluster of related contigs. Finally, these Bruijn graphs were processed to find the path based on the reads and paired reads in the graphs to obtain the transcripts. To comprehensively obtain gene annotation information, genes were compared with six databases, including NR (NCBI non-redundant protein sequences), Gene Ontology (GO), Kyoto Encyclopedia of Genes and Genome (KEGG), eggNOG (evolutionary genealogy of genes: Non-supervised Orthologous Groups), Swiss-Prot, and Pfam, and the annotation situation of each database was counted.

### Differentially expressed genes (DEGs) and enrichment analysis

RSEM estimated gene expression levels for each sample (Li and Dewey, 2011). The gene abundances were calculated and normalized to Reads Per kb per Million reads (RPKM). Three pairwise comparisons were made from RNA-seq data, including inoculation 0 h (CK) vs. inoculation 2 h (GT2), inoculation 0 h vs. inoculation 12 h (GT12), and inoculation 0 h vs. inoculation 24 h (GT24). DEGs was performed using DESeq2 (Love et al., 2014) software between two groups. The genes with the parameter of *P*-value < 0.05 and | log_2_^FoldChange^| > 1 were considered DEGs. All DEGs were mapped to GO terms in the GO database,^1^ gene numbers were calculated for every term, and significantly enriched GO terms in DEGs compared to the genome background were defined by hypergeometric test. The criterion for significant enrichment of GO function was *P*-value < 0.05. Pathway enrichment analysis identified significantly enriched metabolic pathways or signal transduction pathways in DEGs compared with the whole genome background. The corrected *P*-values adopted 0.05 as the threshold, and KEGG pathways meeting the above conditions were defined as significantly enriched pathways in DEGs.

### Quantitative real-time polymerase chain reaction (qRT-PCR) verification

RNA-seq results were validated by selecting six DEGs to examine the consistency of their expression profiles. Total RNAs were extracted from collected *G. lingzhi* materials using the Trizol (Invitrogen, USA) kit according to the manufacturer’s instructions. First-strand cDNAs were synthesized by the PrimeScript™ 1st stand cDNA Synthesis Kit. The internal transcribed spacer (ITS) gene was used as an internal control. Volume for all the reactions was 20 μL; 1 μL cDNA, 10 μL 2 × SYBR real-time PCR (Applied Biosystem, Carlsbad, CA, USA), and 0.4 μL of each primer. The PCR procedure was 5 min at 95°C, followed by 40 cycles of 15 s at 95°C and 30 s at 60°C. Three biological replicates were performed per sample. The formula of 2^–ΔΔCT^ was used to calculate gene relative expression levels.

## Results

### Summary of transcriptome analysis

Four sample sets, each with three biological replicates, were subjected to RNA-seq, and 12 cDNA libraries were generated: GT0-1, GT0-2, GT0-3, GT2-1, GT2-2, GT2-3, GT12-1, GT12-2, GT12-3, GT24-1, GT24-2, GT24-3. Samples were inoculated for 0 h (GT0-1, GT0-2, GT0-3) as a control. The sequencing raw data set has been deposited in National Center for Biotechnology Information Sequence Read Archive database (accession number PRJNA917261). Approximately 4.169∼5.386 million raw reads were produced for each sample, with the percentages of Q20 and Q30 being over 97.64 and 93.75%, respectively. A total of 79.7 Gb of clean data was obtained and the clean data of each sample reached more than 6.0 Gb. A transcriptome database containing 36,870,053 unigene of total length was obtained using Trinity software, with a mean length of 1,850.35 bp and GC content of 57.06%. All unigenes and transcripts obtained by transcriptome assembly were aligned with six major databases (Nr, Swiss-prot, Pfam, COG, GO, and KEGG databases).

### Differentially expressed gene (DEG) analysis in *Ganoderma lingzhi*

**FIGURE 1:**
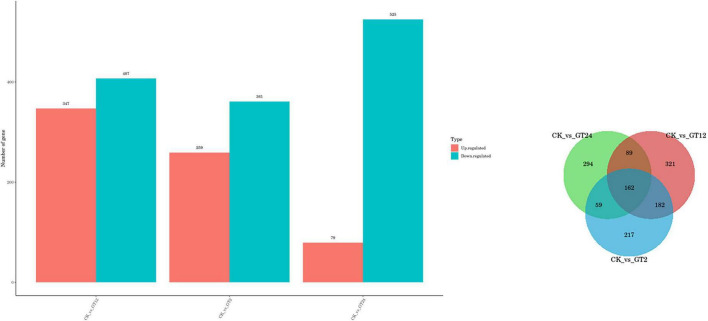
Differentially expressed genes (DEGs) between samples. **Left**: numbers of DEGs compared between two samples (CK vs. GT2, CK vs. GT12, and CK vs. GT24). DEGs are shown in red (up-regulated) and blue (down-regulated). **Right**: Venn diagram analysis of the DEGs in *Ganoderma lingzhi* after inoculation with *Trichoderma hengshanicum*.

Differentially expressed genes in susceptible *G. lingzhi* at different time points were identified using the thresholds *p* < 0.05 and | log_2_^FoldChange^| > 1. In response to the fungal stimulus, a total of 1,978 genes showed differential expression at three-time points after inoculation (Figure 1). A total of 754 (347 up-regulation and 407 down-regulation), 620 (259 up-regulation and 361 down-regulation), and 604 (79 up-regulation and 525 down-regulation) differential genes were observed in CK vs. GT2, CK vs. GT12, and CK vs. GT24, respectively. The Venn diagram (Figure 1) showed that both shared and unique DEGs were identified between different combinations. There were 162 shared DEGs in CK vs. GT2, CK vs. GT12, and CK vs. GT24.

### Functional annotation of differentially expressed genes (DEGs)

**FIGURE 2:**
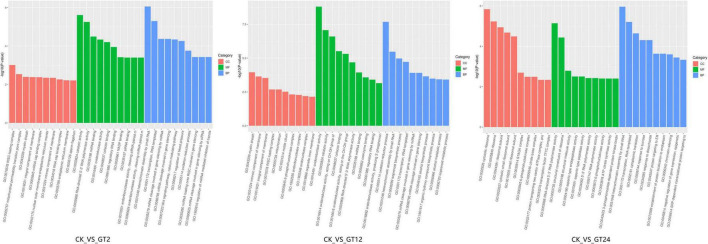
Gene Ontology (GO) functional enrichment analyzes of differentially expressed genes (DEGs).

Gene Ontology classification analysis of DEGs between CK vs. GT2, CK vs. GT12, and CK vs. GT24 was shown in Figure 2. GO had three ontologies, describing the molecular function, cellular component, and biological process of genes. At 2 h after infection, GO enrichment analysis of DEGs showed that 396, 47, and 128 GO terms were identified in biological processes, cellular components, and molecular functions, respectively. The most significant enrichment of DEGs in the biological process ontology was heterochromatin assembly by small RNA, mRNA cleavage involved in gene silencing, Wnt signaling pathway-calcium modulating pathway, and transcription (RNA-templated), whereas RNA-induced silencing complex (RISC)-loading complex and mitochondrial permeability transition pore complex occupied important positions in the cellular component ontology. The most significantly enriched molecular function ontology was catalytic activity, RNA-directed 5′-3′ RNA polymerase activity, miRNA binding, oxidoreductase activity, cofactor binding, regulatory RNA binding, nicotinamide adenine dinucleotide phosphate (NADP) binding, siRNA binding, endoribonuclease activity (cleaving siRNA-paired mRNA), endoribonuclease activity (cleaving miRNA-paired mRNA), oxidoreductase activity (acting on the CH-OH group of donors), monooxygenase activity, and iron ion binding. At 12 h after infection, GO analysis of DEGs revealed 360 entries related to biological processes, 52 cellular components, and 150 molecular functions. According to the GO annotations analysis at Level 2, the most significant enrichment of DEGs was in the oxidation-reduction process, heterochromatin assembly by small RNA, isoprenoid biosynthetic process ontology, transcription, and RNA-templated in the biological process ontology. The most significantly enriched molecular function ontology was oxidoreductase activity, catalytic activity, oxidoreductase activity (acting on CH-OH group of donors), cofactor binding, oxidoreductase activity (acting on the CH-OH group of donors, NAD or NADP as acceptor), RNA-directed 5′-3′ RNA polymerase activity, miRNA binding, coenzyme binding, and regulatory RNA binding, while myelin sheath, an intrinsic component of the membrane and integral component of membrane occupied the important positions in the cellular component ontology. At 24 h after infection, DEGs were divided into 390 functional categories according to 275 biological processes, 34 cellular components, and 81 molecular functions. The most enriched DEGs in the biological processes belonged to heterochromatin assembly by small RNA, cytoplasmic translation, response to sucrose, response to disaccharide and transcription, and RNA-templated. There were 34 terms related to cellular components, among which the most significantly enriched were cytosolic ribosome, ribosome, ribosomal subunit, cytosolic small ribosomal subunit, and small ribosomal subunit; 81 GO terms related to molecular function were identified, of which, RNA-directed 5′-3′ RNA polymerase activity and structural constituent of ribosome were the most significantly enriched metabolic pathways. In the biological process ontology, CK vs. GT2, CK vs. GT12, and CK vs. GT24 had the two most significantly enriched terms, namely, heterochromatin assembly by small RNA and transcription, RNA-templated. RNA-directed 5′-3′ RNA polymerase activity was the most significantly enriched cellular component ontology term in CK vs. GT2, CK vs. GT12, and CK vs. GT24. Five of the most significantly enriched terms in CK vs. GT2 and CK vs. GT12 were related to molecular function ontology, which were catalytic activity, oxidoreductase activity, oxidoreductase activity (acting on the CH-OH group of donors) and regulatory RNA binding, and were also enriched in CK vs. GT24.

**FIGURE 3:**
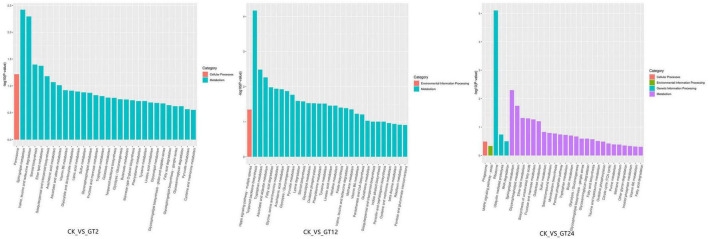
Kyoto Encyclopedia of Genes and Genome (KEGG) pathway enrichment analyzes of differentially expressed genes (DEGs).

To elucidate the main metabolic pathways involved in the DEGs responding to *T. hengshanicum* stress in *G. lingzhi*, the KEGG enrichment analysis was conducted in CK vs. GT2, CK vs. GT12, and CK vs. GT24. The top 20 KEGG pathways with the lowest false discovery rate (FDR) values are shown in Figure 3. The greater the richness factor, the greater the enrichment. DEGs annotated 93 metabolic pathways in CK vs. GT2, CK vs. GT12, and CK vs. GT24. Sphingolipid metabolism, ether lipid metabolism, steroid biosynthesis, and valine, leucine and isoleucine degradation were the most significantly enriched metabolic pathways in CK vs. GT2. Terpenoid backbone biosynthesis, glycine, serine and threonine metabolism, tryptophan metabolism, ascorbate, and aldarate metabolism, fatty acid degradation, arachidonic acid metabolism, glycolysis/gluconeogenesis, pyruvate metabolism, lysine degradation, steroid biosynthesis, glycerolipid metabolism, oxidative phosphorylation, phenylalanine metabolism, tyrosine metabolism, linoleic acid metabolism, histidine metabolism, valine, leucine and isoleucine degradation, folate biosynthesis, methane metabolism, and hippo signaling pathway-multiple species were the most significantly enriched metabolic pathways in CK vs. GT12. The most significantly enriched metabolic pathways in CK vs. GT24 belonged to the ribosome, sphingolipid metabolism, glycerophospholipid metabolism, ether lipid metabolism, and biosynthesis of unsaturated fatty acids. Valine, leucine and isoleucine degradation was significantly enriched in CK vs. GT2, and CK vs. GT12, and also enriched in CK vs. GT24. There were two significant metabolic pathways in CK vs. GT2 and CK vs. GT24: sphingolipid metabolism and ether lipid metabolism, which were also enriched in CK vs. GT12.

### qRT-PCR analysis

**FIGURE 4:**
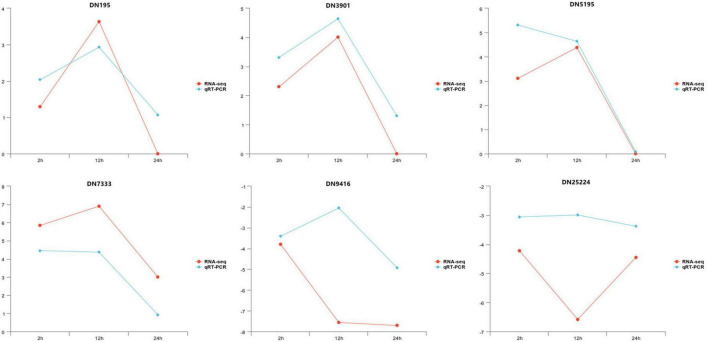
The relative expression level change of six selected genes from differentially expressed genes (DEGs) by quantitative real-time polymerase chain reaction (qRT-PCR).

To confirm the reliability of the generated RNA-seq data, the expression of six DEGs was analyzed using qRT-PCR validation. Genescloud tools^2^ were used to visualize the results (Figure 4). Although the magnitude of differences in expression detected by qRT-PCR was not identical to those of DEGs detected by the RNA-seq results from the samples at three infection time points, the direction of the change in DEG expression was consistently using the two approaches, indicating that the results of transcriptome sequencing were highly reliable.

### Sphingolipid metabolism

**FIGURE 5:**
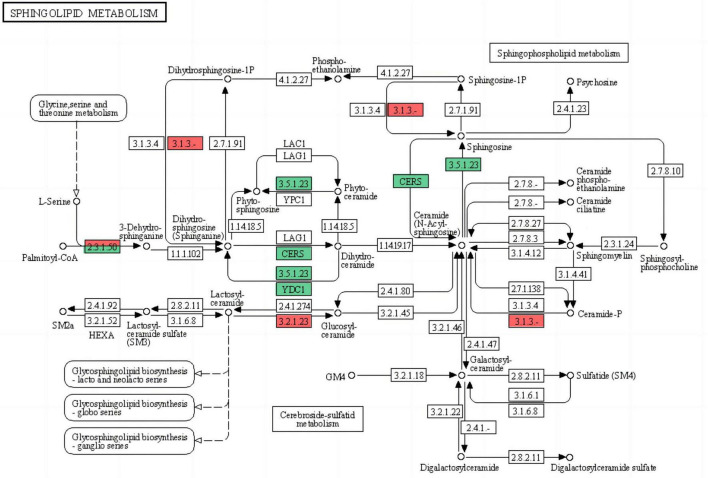
Differentially expressed genes (DEGs) comparison between two samples (CK vs. GT2, CK vs. GT12, and CK vs. GT24) mapped to the sphingolipid metabolism (map00600; red for up-regulated, green for down-regulated).

**FIGURE 6:**
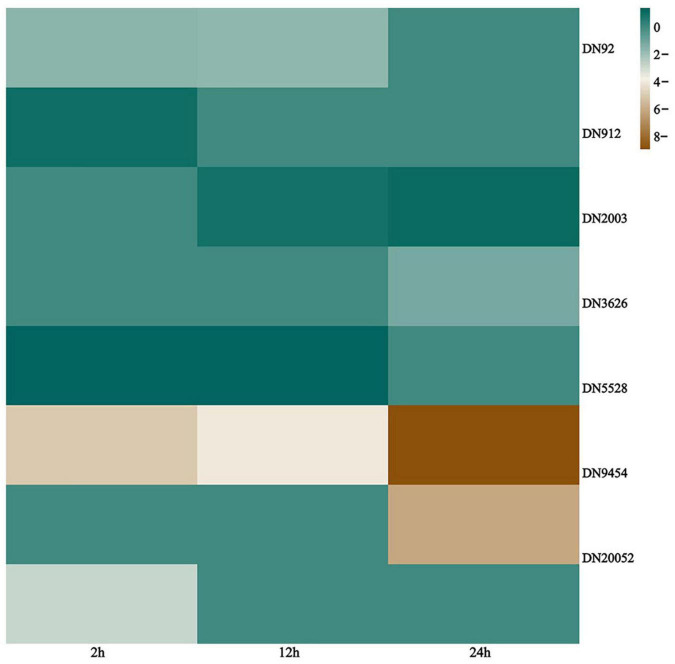
Heatmaps of differentially expressed genes (DEGs) involved in sphingolipid metabolism pathways. The log_2_^Foldchange^ was colored using Genescloud tools (green for up-regulated, brown for down-regulated).

The basic structure of biomembranes comprises various lipids, such as glycerophospholipids, sphingolipids, and sterols, and proper homeostasis of the composition of these lipids in biomembranes is extremely important for the maintenance of multiple cellular functions (Tani and Funato, 2018). Sphingolipids are essential biomembrane lipids for eukaryotic organisms. They commonly have a long-chain base (LCB) backbone. Ceramide (Cer), the hydrophobic portion of sphingolipids, comprises an LCB and a fatty acid (Dickson et al., 2006; Tani and Funato, 2018). In this study, the sphingolipid metabolism pathway of *G. lingzhi* was significantly enriched after *T. hengshanicum* infection (Figure 5). As shown in the Figure 6, two genes were up-regulated and three genes were down-regulated in the phospholipid metabolic pathway at 2 h after infection. At 12 h after infection, one gene was up-regulated and two genes were down-regulated in the phospholipid metabolic pathway. At 24 h after infection, one gene was up-regulated and four genes were down-regulated in the phospholipid metabolic pathway. This indirectly showed that *T. hengshanicum* infection would affect sphingolipid metabolism in *G. lingzhi*.

### Pathogenesis-related proteins

**FIGURE 7:**
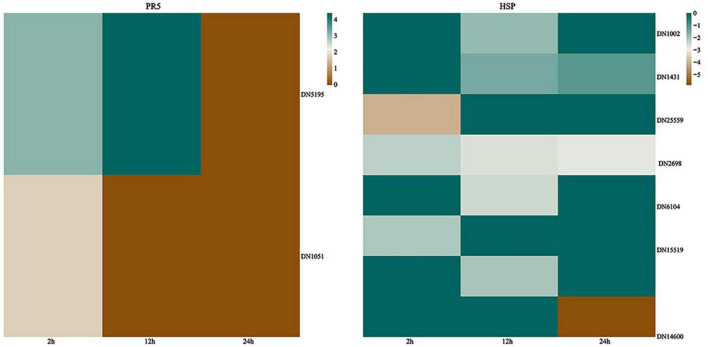
**Left**: Heatmaps of differentially expressed genes (DEGs) encoding thaumatin-like proteins (TLPs); **right**: Heatmap of DEGs encoding heat shock proteins (HSPs). The log_2_^Foldchange^ was colored using Genescloud tools (green for up-regulated, brown for down-regulated), each horizontal row represents a DEG with its gene ID.

Pathology-associated proteins are a class of proteins induced by plants in pathological or pathology-associated environments, initially detected from tobacco mosaic virus infection in tobacco leaves (Li, 2020). Pathogenesis-related (PR) proteins are comprised of 17 families that are normally expressed at low levels in healthy tissues but rapidly accumulate to significant levels in response to biotic or abiotic stress (Van Loon and Van Strien, 1999; Van Loon et al., 2006; Zhu et al., 2017). It was known that the timing of PR gene expression was a crucial determinant of pathogenesis. The accumulation of PR proteins is usually associated with systemic acquired resistance to a wide range of pathogens. *G. lingzhi* PR genes were induced in response to *T. hengshanicum* infection in our experiment. Our results showed that *G. lingzhi* up-regulated PR thaumatin-like proteins (TLPs) (PR-5s) after infection with *T. hengshanicum*. The PR-5s genes were up-regulated 8.6-fold at 2 h post-infection (Figure 7).

Heat shock proteins (HSPs) are a subset of molecular chaperones, best known because they are rapidly induced in large numbers by stress (Neumann et al., 1994; Wang et al., 2004; Scarpeci et al., 2008). These proteins are implicated in a wide variety of cellular processes as molecular chaperons, including the protection of the proteome from stress, the folding and transport of newly synthesized polypeptides, the activation of proteolysis of misfolded proteins, and the formation and dissociation of protein complexes, plays a pivotal role in the protein quality control system, ensuring the correct folding of proteins, the re-folding of misfolded proteins, and controlling the targeting of proteins for subsequent degradation. The results showed that HSPs in the *G. lingzhi* displayed a range of responses to *T. hengshanicum*. At the three time points of infection, the HSPs genes of *G. lingzhi* were down-regulated (Figure 7).

## Discussion and conclusion

*Ganoderma lingzhi* has high application value in the prevention and treatment of nephritis, hypertension, and bronchitis, and has remarkable antitumor properties, and is deeply loved by people (Wu et al., 2011; Nie et al., 2013; Vitak et al., 2015; Paterson, 2016; Yan et al., 2019). With the rapid expansion of *G. lingzhi* cultivation, however, green mold has become one of the severe diseases threatening the production of *G. lingzhi*. After the fruiting bodies were infected with *Trichoderma* spp., lesions appeared and were covered by green mycelium. The infected fruiting bodies became deformed and withered as the disease progressed (Yan et al., 2019; Cai et al., 2020). Therefore, it is important to use disease-resistant variety to control disease that damage the quantity and quality of *G. lingzhi*. Currently, there are many studies on the interaction between plants and pathogens. In response to external biotic stresses, plants induce a series of immune responses, including the production of physical barriers (keratin, wax, lignin, and special stomatal structures), chemical barriers (secondary metabolites with antimicrobial properties), and molecular responses (hypersensitivity, production of reactive oxygen species, and expression of pathogen-associated genes) (Ding and Yang, 2016). Therefore, it is inevitable that macrofungi will also produce various defensive responses to resist the pathogen infection. For breeding disease-resistant *G. lingzhi*, it is necessary to have information on genetic variation in the hosts reaction to disease infestation. In this research, we studied the transcription of *G. lingzhi* at 2, 12, and 24 h after infection by *T. hengshanicum*, and explored the resistance genes and metabolic pathways induced by *T. hengshanicum*.

In this study, we selected six DEGs related to the *T. hengshanicum* infection of *G. lingzhi* for qRT-PCR verification. Our results revealed the same trend in DEG expression as established by transcriptome sequencing results, indicating high reliability of the transcriptome sequencing results in reflecting the proper expression levels of genes in *G. lingzhi* infected by *T. hengshanicum*. The six DEGs verified by qRT-PCR were the TLPs (PR-5s) gene, the phenylalanine ammonia-lyase gene, the cyanide hydratase gene, the Beta-1,3-glucan binding protein, the polyketide synthase gene, and the 6-phosphofructokinase gene. TLPs have antifungal and osmotic adjustment activities or act as an elicitor of other antifungal proteins and play an important role in the growth and development of the host and the process of stress resistance (Menu-Bouaouiche et al., 2003; Guo et al., 2016; Faillace et al., 2019; Sun et al., 2020; Liu Y. et al., 2021; Liu et al., 2022). Previous studies have shown that PR-5s is involved in the plant defense response induced by diseases and insects (Hou et al., 2018). For example, overexpression of the rice TLPs gene significantly increased the resistance of rice, and wheat to related diseases (Chen et al., 1999; Datta et al., 1999). As a result, up-regulation of PR5 expression at 2 h after infection benefits *G. lingzhi* in preventing a violation by *T. hengshanicum*. The phenylalanine ammonia-lyase gene was also up-regulated after infection. As the first rate-limiting enzyme in the phenylpropanoid metabolism pathways, phenylalanine ammonia-lyase (PALs) can catalyze the deamination of L-phenylalanine to form *trans*-cinnamic acid, which is a precursor of lignin, salicylic acid (SA), flavonoids, phytoalexins, as well as bioactive phenolamides *via* specific branch pathways, playing important roles in plant growth, development, and stress responses (Dixon et al., 2002; Zhang and Liu, 2015; You et al., 2020). For example, enhanced deposition of lignin can reinforce the plant cell wall, providing a structural barrier to pathogen spread, and the toxic phenolic precursors produced during lignin biosynthesis or polymerization can directly inhibit pathogen multiplication and movement (Tonnessen et al., 2015). The up-regulation of phenylalanine ammonia-lyase gene expression in *G. lingzhi* might activate the phenylpropanoid metabolic pathway in *G. lingzhi* and then produce some or specific related secondary metabolites to resist the infection of *T. hengshanicum*. Moreover, the Beta-1,3-glucan binding protein (LGBP) molecule was reported to have antibacterial, anti-biofilm, anti-inflammatory, and antioxidant properties (Iswarya et al., 2017). Many studies have shown that β-1,3-glucan binding proteins are host pattern recognition receptors (PRRs) that recognize conserved surface ligands in microorganisms designed the pathogen-associated molecule patterns (PAMPs) and have a strong affinity toward the –glucans present on the surface of bacteria and fungi, thereby activating the prophenoloxidase (proPO) activating system to elicit the invertebrate innate defense system (Zhang et al., 2016; Anjugam et al., 2017; Li S. S. et al., 2022). The results of this study revealed that *T. hengshanicum* infection of *G. lingzhi* not only up-regulated β-1,3-glucan binding proteins but also tyrosinase, and the infection site of *G. lingzhi* turned brown 24 h after *T. hengshanicum* infection, which could be due to activation of the prophenoloxidase system in *G. lingzhi*, resulting in the production of melanin or other secondary metabolite deposition. Intermediate sphingolipid metabolic pathways are important signal molecules closely related to cell growth, apoptosis, differentiation, senescence, stress resistance, and signaling transduction (Li J. et al., 2022). Free sphingosine binds to different substances in organisms to form Cer, sphingomyelin, and glycosphingolipids (Dickson et al., 2006; Harrison et al., 2018). In the study of *Saccharomyces cerevisiae*, long-chain sphingolipid bases are signaling molecules that regulate growth, responses to heat stress, cell wall synthesis and repair, endocytosis, and dynamics of the actin cytoskeleton (Dickson et al., 2006). In plants, sphingolipids were not only the main components of the plant plasmalemma, tonoplast membrane, and intima but also participated in various plant stress responses as the second messenger of plant defense mechanisms (Markham et al., 2013; Shan et al., 2019). Changes in sphingolipid content and sphingolipid/phosphorylated derivative balance in plants infected with microorganisms regulated plants to produce a resistance response (Bi et al., 2014; Yanagawa et al., 2017). Therefore, in the interaction between *G. lingzhi* and *T. hengshanicum*, *G. lingzhi* sphingolipids might be the signal molecules that could induce resistance *G. lingzhi*.

These data provided a better understanding of the mechanisms and identified potential DEGs involved in the early disease defenses of *G. lingzhi* against *T. hengshanicum*. These DEGs could be used as a screening indicator for identifying or selecting strains with high-disease-resistant properties. However, the results of this study were slightly different from those of plant-pathogen interactions. No DEGs related to plant hormones (JA, SA, and ABA, etc.) signaling transduction pathways were found in *G. lingzhi* infected by *T. hengshanicum*. In plants, SA and JA were endogenous plant hormones, that could induce the expression of pathogenicity-related proteins and systemic acquired resistance in plants, and were also recognized as a signal of plant responses to abiotic and biotic stresses. In *A. bisporus*, after *P. tolaasii* infection, JA biosynthesis and signaling transduction pathways were significantly enriched, and JA content was also detected to increase (Ma et al., 2021). Furthermore, no differential changes in genes related to cell wall synthesis were found in this study. In plants, the cuticle is the first cell wall layer encountered by a pathogen, plant pathogens must overcome the physical barrier presented by the cuticle and plant cell wall, so that the plant cell wall undergoes very large cell wall remodeling. Therefore, the changes in genes related to plant hormones and cell wall synthesis enzymes must be further studied.

In conclusion, transcriptomic analysis detected 620, 754, and 604 DEGs at 2, 12, and 24 h after infection with *T. hengshanicum*. Transcriptome sequencing indicated that there were 162 DEGs at three infection time points, containing 15 up-regulated DEGs and 147 down-regulated DEGs. After *G. lingzhi* was infected by *T. hengshanicum*, most of the DEGs were down-regulated at three-time points, indicating that *G. lingzhi* may resist the infection of *T. hengshanicum* mainly by down-regulating gene expression. Resistance-related genes TLPs (PR-5s) gene, phenylalanine ammonia-lyase gene, Beta-1,3-glucan binding protein were significantly up-regulated. At the three-time points of infection, the HSPs genes of *G. lingzhi* were down-regulated. The down-regulation of HSPs genes led to the inhibition of HSP function, which may compromise the HSP-mediated defense signaling transduction pathway, leading to *G. lingzhi* susceptibility. We performed GO and pathway enrichment analysis of DEGs at 2, 12, and 24 h for susceptible *G. lingzhi*, respectively. Four different gene sets were enriched for GO classification and KEGG enrichment, the main GO enrichment included heterochromatin assembly by small RNA and transcription, RNA-templated, RNA-directed 5′-3′ RNA polymerase activity, catalytic activity, oxidoreductase activity, oxidoreductase activity (acting on CH-OH group of donors), and regulatory RNA binding and the enriched pathways included sphingolipid metabolism, ether lipid metabolism, and valine, leucine and isoleucine degradation pathway. Although the *T. hengshanicum* pathogens induced resistance in *G. lingzhi*, such resistance could not completely prevent pathogen invasion, thereby showing disease symptoms. In conclusion, our results revealed the DEGs and metabolic pathways in the early defense response of *Trichoderma* spp. and provided a theoretical basis for the breeding of resistant strains of *G. lingzhi*.

## Data availability statement

The data presented in the study are deposited in the National Center for Biotechnology Information Sequence Read Archive repository, accession number PRJNA917261.

## Author contributions

TW wrote the manuscript. TW and CZ carried out experiments and collecting specimens. JX revised the manuscript and designed experiments. XL revised the manuscript and submitted the transcriptome data. All authors contributed to the article and approved the submitted version.

## Footnotes

1
http://geneontology.org/

2
https://www.genescloud.cn
